# Estradiol and vitamin D exert a synergistic effect on preventing osteoporosis via the miR-351-5p/IRS1 axis and mTOR/NFκB signaling pathway

**DOI:** 10.1038/s41598-025-02808-z

**Published:** 2025-05-28

**Authors:** Xiaoyan Dai, Changcun Liu, Wenkai Bi, Guiwen Zheng, Kuan Lv, Zhiming Xia

**Affiliations:** 1 Department of Otolaryngology, Shandong Provincial Hospital Affiliated to Shandong First Medical University, Jinan, Shandong China; 2 Department of Nuclear Medicine, Shanghai General Hospital, Shanghai Jiao Tong University School of Medicine, Shanghai, P.R. China; 3 Department of Nuclear Medicine, Shandong Provincial Hospital Affiliated to Shandong First Medical University, Jinan, Shandong China

**Keywords:** Osteoporosis, Estradiol, Vitamin D, miR-351-5p, IRS1, mTOR/NFκB signaling pathway, Molecular biology, Diseases, Medical research

## Abstract

This study aimed to investigate the antiosteoporotic effects and regulatory mechanisms of estradiol (E2) and vitamin D. MC3T3-E1 cells were treated with E2, vitamin D, or their combination, followed by a systematic assessment of cell proliferation and osteogenic differentiation capacity across the treatment groups. Subsequently, miRNA sequencing was performed to analyze differentially expressed miRNAs between the control and E2&vitamin D groups. The target relationship between miR-351-5p and IRS1 was validated, and the effects of the miR-351-5p/IRS1 axis on osteogenesis and mTOR/NFκB signaling pathway were determined after combination treatment. Additionally, an ovariectomized (OVX) osteoporosis mouse model was established to ‌systematically examine‌ the effects of E2, vitamin D, and their combination on osteoporosis and mTOR/NFκB signaling pathway. E2 and vitamin D synergistically promoted MC3T3-E1 cell proliferation and osteogenic differentiation. miR-351-5p was identified through miRNA sequencing analysis. miR-351-5p was downregulated in MC3T3-E1 cells after E2 and vitamin D combination treatment, and its overexpression partially reversed the effect of the combination treatment on osteogenesis. IRS1 was a target of miR-351-5p. When overexpressed, IRS1 partially mitigated the impact of miR-351-5p overexpression on osteogenesis and mTOR/NFκB signaling pathway under the combination treatment. Furthermore, in vivo experiments demonstrated that E2 and vitamin D could synergistically prevent osteoporosis in OVX mice by inhibiting the mTOR/NFκB signaling pathway. In conclusion, E2 and vitamin D exhibited a synergistic effect in preventing osteoporosis through the miR-351-5p/IRS1 axis and mTOR/NFκB signaling pathway. E2 and vitamin D combination treatment could be a potential anti-osteoporotic strategy for osteoporosis treatment.

**Supplementary Information:**

The online version contains supplementary material available at 10.1038/s41598-025-02808-z.

## Background

Osteoporosis is a prevalent global bone disorder characterized by decreased bone mineral density (BMD), which makes affected bones more susceptible to fractures^1,2^. According to a study conducted by the European Union, the number of osteoporotic fractures is estimated to rise by 4.5 million a year^3^. Despite the development of numerous drugs for osteoporosis treatment, including calcitonin, bisphosphonates, and molecular targeted drugs, many of these medications have significant adverse effects and are considered unsuitable for prolonged use^4^. Therefore, it is crucial to explore effective therapeutic drugs and elucidate the key mechanisms underlying osteoporosis.

Vitamin D is critical for maintaining bone health by regulating calcium absorption and is essential for the prevention and management of osteoporosis^5^. Nonetheless, relying solely on vitamin D supplements to effectively manage the risk of osteoporosis or fractures has been considered insufficient by prior reports^6^. Additionally, disturbances in bone metabolism balance are linked to estrogen deficiency, which is commonly observed in postmenopausal women^7^. Estradiol (E2) is used in hormone replacement therapy for osteoporosis^8^. Vitamin D exerts its effect on osteoporosis through interactions with sex hormones, such as total testosterone^9^. Deficiencies in both vitamin D and E2 have been found have a synergistic effect on metabolic syndrome in postmenopausal Chinese women^10^. However, the extent to which vitamin D and E2 synergistically prevent osteoporosis and the underlying regulatory mechanisms remain largely unknown.

MicroRNAs (miRNAs) are endogenous, non-coding small RNAs that play crucial roles in various biological processes within the bone tissue, including cell proliferation and differentiation^11^. They exert their regulatory functions by binding to the 3’ UTR of target mRNAs, thereby modulating gene expression post-transcriptionally^12^. Aberrant regulation of miRNAs has been implicated in the development of bone-related disorders, such as osteoporosis^13,14^. For example, miR-196b-5p inhibits osteoblast differentiation and regulates bone homeostasis by targeting SEMA3A^15^. Additionally, miR-505 modulates the osteogenic differentiation of MC3T3-E1 cells by targeting RUNX2^16^. However, the key miRNAs mediating the anti-osteoporotic effects of vitamin D and E2 remain elusive.

The present study aimed to investigate the anti-osteoporotic effects of E2 and vitamin D in MC3T3-E1 cells. Subsequently, miRNA sequencing was conducted to identify the key miRNAs associated with E2 and vitamin D combination treatment. The target relationship between miR-351-5p and IRS1 was also explored, and the effects of miR-351-5p/IRS1 axis on the osteogenesis and mTOR/NFκB signaling pathway were determined after the combination treatment. Additionally, the effects of E2, vitamin D, and their combination on osteoporosis were validated in vivo. Our findings provide novel insights for osteoporosis treatment.

## Methods

### Cell lines and osteogenic induction

The pre-osteoblastic MC3T3-E1 cell line was purchased from Shanghai Wenye Biotechnology Co., Ltd. (Shanghai, China). MC3T3-E1 cells were cultured in the osteogenic induction medium comprising 10% fetal bovine serum (Gibco, USA), 10^− 7^ M dexamethasone (Sigma, USA), 10 mM β-glycerophosphate (Sigma), and 50 µg/mL ascorbic acid (Sigma-Aldrich) for 14 d.

### Cell treatment

To evaluate the effect of E2 and vitamin D, MC3T3-E1 cells were treated with various concentrations of E2 (10^− 5^, 10^− 6^, 10^− 7^, 10^− 8^, 10^− 9^, and 10^− 10^ M) and vitamin D (10^− 6,^ 10^− 7^, 10^− 8^, 10^− 9^, 10^− 10^, 10^− 11^, and 10^− 12^ M).

### Cell transfection

MC3T3-E1 cells were seeded in 96-well plates at a density of 5 × 10^4^ cells/mL per well. Cells were transfected with 50 nM miR351-5p mimics and miRNA-negative control (miR-NC) using Lipofectamine 2000 (Invitrogen). Cells were collected at 48 h after transfection.

For IRS1 overexpression and knockdown experiments, the oe-IRS1 and si-IRS1 sequences were integrated into the pLKO.1 lentiviral vector. To generate high-titer lentiviral particles, the lentiviral vector was co-transfected into 293T cells with packaging plasmids using Lipofectamine 2000. At 48 h after transfection, MC3T3-E1 cells (2 × 10^5^/mL) were plated into 6-well plates and infected with lentiviral particles at a multiplicity of infection (MOI) of 1 × 10^8^ TU/mL. At 72 h after transfection, the cells were harvested.

### Real-time PCR

Total RNA was extracted using RNAios Plus reagent (TaKaRa, Japan) and reverse-transcribed to cDNA using a cDNA synthesis kit (CWBIO, Beijing, China). Quantitative real-time PCR was performed using a SYBR Premix Ex Taq kit (Shanghai Meixuan Biotechnology Co., LTD, China) on a CFX Connect 96 system (BIO-RAD CFX Manager, USA). The amplification protocol was as follows: 95 °C, 5 min; 95 °C, 15 s and 60 °C, 30 s for 40 cycles; and 60 °C, 2 min. U6 or GAPDH was used as the internal reference gene. The primer sequences are provided in Table 1. Data analysis was conducted using the comparative Ct method (2^−ΔΔCt^).

**Table 1:** Primer sequences (5 ‘ to 3 ‘ ).

Gene	Forward	Reverse
miR-351-5p	GCGCTCCCTGAGGAGCCCTTTG	TGCAGGGTCCGAGGTAT
U6	CTCGCTTCGGCAGCACA	AACGCTTCACGAATTTGCGT
IRS1	ATGCCAATCACTCGAATGCG	TTGTATCGGCCTGTGTGAATG
GAPDH	GACAGCCGCATCTTCTTGTG	AATCCGTTCACACCGACCTT

### CCK-8 assay

The cells were digested, collected, and resuspended. Subsequently, the cell suspension (5000 cells) was inoculated into a 96-well plate. The plate was then cultured for 12 h at 37 ℃ in a 5% CO_2_ incubator, after which the cells were subjected to different treatments. After incubation for 48 h, 10 µL of CCK-8 solution (Servicebio) was added into each well, and the plate was incubated in the same incubator conditions (37 °C, 5% CO_2_) for 1–3 h. The absorbance (OD value) at 450 nm was measured using a microplate reader ( Pulang New Technology Co., Ltd., Beijing, China).

### Alizarin red staining

MC3T3-E1 cells were cultured in an osteogenic induction medium for 21 d. The cells were rinsed twice with PBS, fixed with 4% paraformaldehyde for 15 min at room temperature, and stained with Alizarin Red S solution (Serbicebio, Beijing, China) for 5 min. After washing, cells were observed and photographed under a microscope (LW300LFT-LED; Shanghai Measurement Dimension Photoelectric Technology Co., Ltd.).

### Alkaline phosphatase (ALP) activity assay

MC3T3-E1 cells were lysed with ice-cold lysis buffer, and the supernatants were collected and quantified using a BCA Protein Assay Kit (Thermo Scientific, USA). The activity of ALP was determined using an ALP assay kit (Nanjing Jiancheng Bioengineering Institute, Nanjing, China). The OD density at 520 nm was measured using a microplate reader (Beijing Pulang New Technology Co., Ltd.).

### Enzyme linked immunosorbent assay (ELISA)

MC3T3-E1 cells were lysed with ice-cold lysis buffer, and the supernatants were collected and quantified using a BCA Protein Assay Kit (Thermo Scientific, USA). The BGP and CTX contents in the cell supernatants were detected using a Mouse BGP ELISA kit and Mouse CTX ELISA kit (Shanghai Wenye Biotechnology Co., Ltd.), respectively, following the manufacturer’s instructions. The OD density at 450 nm was determined using a microplate reader (Beijing Pulang New Technology Co., Ltd.).

### MiRNA sequencing

Total RNA was extracted from MC3T3-E1 cells in the control group and E2&vitamin D group (combination treatment with the optimal concentration of E2 and vitamin D) using TRIzol^®^ Reagent (Invitrogen, MA, USA). After determining the quality of total RNA, library construction was performed using the TruseqTM Small RNA Sample Prep Kit (Illumina, USA). Libraries were sequenced using Hiseq2000 Truseq SBS Kit v3-HS (50 cycles) (Illumina). For raw reads, quality control was conducted using the Fastx-Toolkit (http://hannonlab.cshl.edu/fastx_toolkit/) to remove adapters and poor quality bases. Clean reads were aligned with the Rfam 11.0 database^17^ using BLAST (version 2.3.0) and mapped to the reference genome using Bowtie (version 1.2.11)^18^. After obtaining the expression data, differentially expressed miRNAs were analyzed using DEGseq2. A heat map of the differentially expressed miRNAs in each sample was drawn using the heatmap2 package.

### Prediction of miR351-5p target genes

The target gene of miR351-5p was predicted using the MiRanda database^19^. Predictive complementary sequences of miR-351-5p and IRS1 were explored using TargetScan.

### Dual-luciferase reporter gene assay

The wild-type (wt)-IRS1 or mutant-type (mut)-IRS1 3’UTR sequences, which contain a putative targeting site for miR351-5p, were amplified and inserted into the psiCHECK2 vector (Promega, Madison, WI, USA) to construct luciferase reporter gene plasmids. Next, 293 T cells (Shanghai Wenye Biotechnology Co., LTD) were cultured in 6-well plates. Luciferase reporter gene plasmids (wt-IRS1 or mut-IRS1 plasmids) and miR351-5 mimics or miR-NC Cells were transfected into cells using lipofectamine 2000 kit (Invitrogen) and incubated for 24 h in an incubator at 37 ℃. Subsequently, firefly and Renilla luciferase activities were detected using a Dual-Luciferase Reagent Assay Kit (Beyotime, Shanghai, China).

### Establishment of ovariectomized (OVX) mouse model of osteoporosis and grouping

Forty 3-month-old healthy female C57 mice (Shanghai SLAC Laboratory Animal Co., Ltd.) were randomly divided into sham, OVX, OVX + E2, OVX + vitamin D, and OVX + E2&vitamin D groups, with eight mice in each group.

An OVX mouse model of osteoporosis was established as follows: mice were anesthetized with 1% pentobarbital sodium solution (50–70 mg/kg, Merck). After anesthesia, the hair in the lumbar spine area of mice was shaved, and the mice were fixed in the prone position on the surgical table. Under sterile conditions, a 2.0 cm incision was made along the lumbar spine, approximately 1–2 cm from the left and right sides. The skin and subcutaneous fascia were dissected sequentially. The ovaries were ligated using a sterile cotton thread, and the bilateral ovaries and adjacent adipose tissues were excised. The surgical incision was sutured, and the wound surface was coated with antibiotics. Subsequently, the mice were placed on a thermostatic table and allowed to recover from anesthesia. In the sham group, only an equivalent mass of the adipose tissue surrounding the ovaries was removed.

Drug administration commenced at 4 weeks after surgery and continued for 4–8 weeks. In the OVX + E2 group, E2 (0.25 mL/kg/d 10 µg/0.1 mL) was administered intraperitoneally once daily in OVX mice. In the OVX + vitamin D group, OVX mice were intraperitoneally injected with vitamin D (6 IU/g/d) at a volume of 0.2 mL per injection. The OVX + E2&vitamin D group received E2 and vitamin D simultaneously. Normal saline was administered to the model and sham surgery groups. After the experiments, mice were anesthetized with ketamine and xylazine and euthanized. Serum was collected from mice to detect ALP activity and BGP and CTX contents. Mouse femur bones were collected for hematoxylin and eosin (HE) staining, von Kossa staining, CT, and western blot assays.

This study was approved by the Animal Ethics Committee of our hospital, and all animal experiments were performed in compliance with the Guide for the Care and Use of Laboratory Animals.

### Micro-computed tomography (microCT) analysis

The femur was scanned using a micro-CT scanner (PINGSENG Healthcare Inc., Kunshan, China). Then, the 3D images of the bone were reconstructed and analyzed using the Avatar3 software (PINGSENG Healthcare Inc.). Bone morphology-related parameters of the femur, including Bone volume/Total volume (BV/TV) and Trabeculae thickness (Tb. Th), Trabeculae Spacing (Tb. Sp) and Trabeculae Number (Tb. N), were analyzed.

### HE staining

Femur tissues were fixed with 4% paraformaldehyde, decalcified in 10% EDTA solution, embedded in paraffin wax, and sectioned into slices (4–7 μm). After dewaxing in dimethylbenzene and hydration with graded ethanol, the sections were subjected to HE staining. Images were captured using a microscope (LW300LFT-LED; Shanghai Measurement Dimension Photoelectric Technology Co., Ltd.).

### Von Kossa staining

Paraffin-embedded sections were dewaxed in dimethylbenzene and hydrated using a graded ethanol series. The sections were stained with Von Kossa solution (Servicebio) and exposed to a UV lamp for 4 h. Following counterstaining with hematoxylin dye (Sigma), images were captured using a microscope, and black-stained spots were regarded as calcium deposits.

### Western blot assay

The cells were lysed in RIPA lysis buffer on ice. After thawing the animal tissue on ice, the tissue block was washed with pre-cooled PBS 2–3 times, placed into the corresponding homogenization tube, and RIPA lysis buffer was added to lyse the tissue. Protein supernatants were collected via centrifugation at 12,000 rpm for 5 min and quantified using a BCA kit (Beyotime). The proteins were subjected to 12% SDS-PAGE and transferred to PVDF membranes. The membranes were blocked with 5% BSA and incubated with primary antibodies to GAPDH (1:1000, BBI Life Sciences, Shanghai, China), IkB (1:1000, Proteintech, Rosemont, IL, USA), p-IkB (1:1000, Zenbio, Durham, NC, USA), mTOR (1:1000, Proteintech), p-mTOR (1:1000, Proteintech), NFkB (1:1000, Proteintech), and p-NFkB (1:1000, Zenbio) overnight at 4 ℃. After washing, the membranes were incubated with horseradish peroxidase-labeled goat anti-mouse/rabbit secondary antibody (1:5000, BBI Life Sciences) at room temperature for 2 h. The membranes were visualized using ECL detection reagent (Millipore, USA). The bands were quantified using ImageJ software.

### Statistical analysis

All experiments were conducted three times, and the data obtained were presented as mean ± standard deviation (SD). Differences among groups were analyzed via one-way ANOVA using GraphPad software version 5.0 (GraphPad Prism, San Diego, CA, USA). Statistical significance was set at *P* < 0.05.

## Results

### Successful osteogenic differentiation of MC3T3-E1 cells

**Fig. 1:**
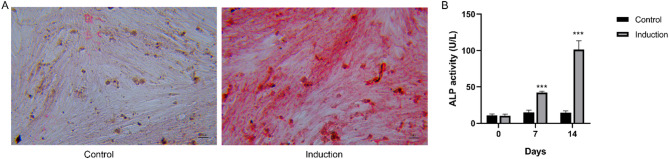
The osteogenic differentiation of MC3T3-E1 cells. A: Alizarin red staining showed the osteogenic differentiation of MC3T3-E1 cells after 14 days of induction. B: The ALP activity of MC3T3-E1 cells after 7 and 14 days of induction. *** *P* < 0.001 compared to 0 days. Each value represents the average of triplicate measurements (*n* = 3).

To induce osteogenic differentiation, MC3T3-E1 cells were cultured in an osteogenic induction medium for 14 d. Alizarin Red staining showed that the cells exhibited a significant accumulation of red-calcified nodules containing mineral salts (Fig. 1A), demonstrating the successful differentiation of MC3T3-E1 cells into osteoblasts. Moreover, ALP activity showed a statistically significant increase at both the 7- and 14-d induction time points (*P* < 0.001; Fig. 1B), quantitatively confirming the osteogenic differentiation potential of these cells.

### E2 and vitamin D could synergistically promote MC3T3-E1 cell proliferation and osteogenic differentiation

**Fig. 2:**
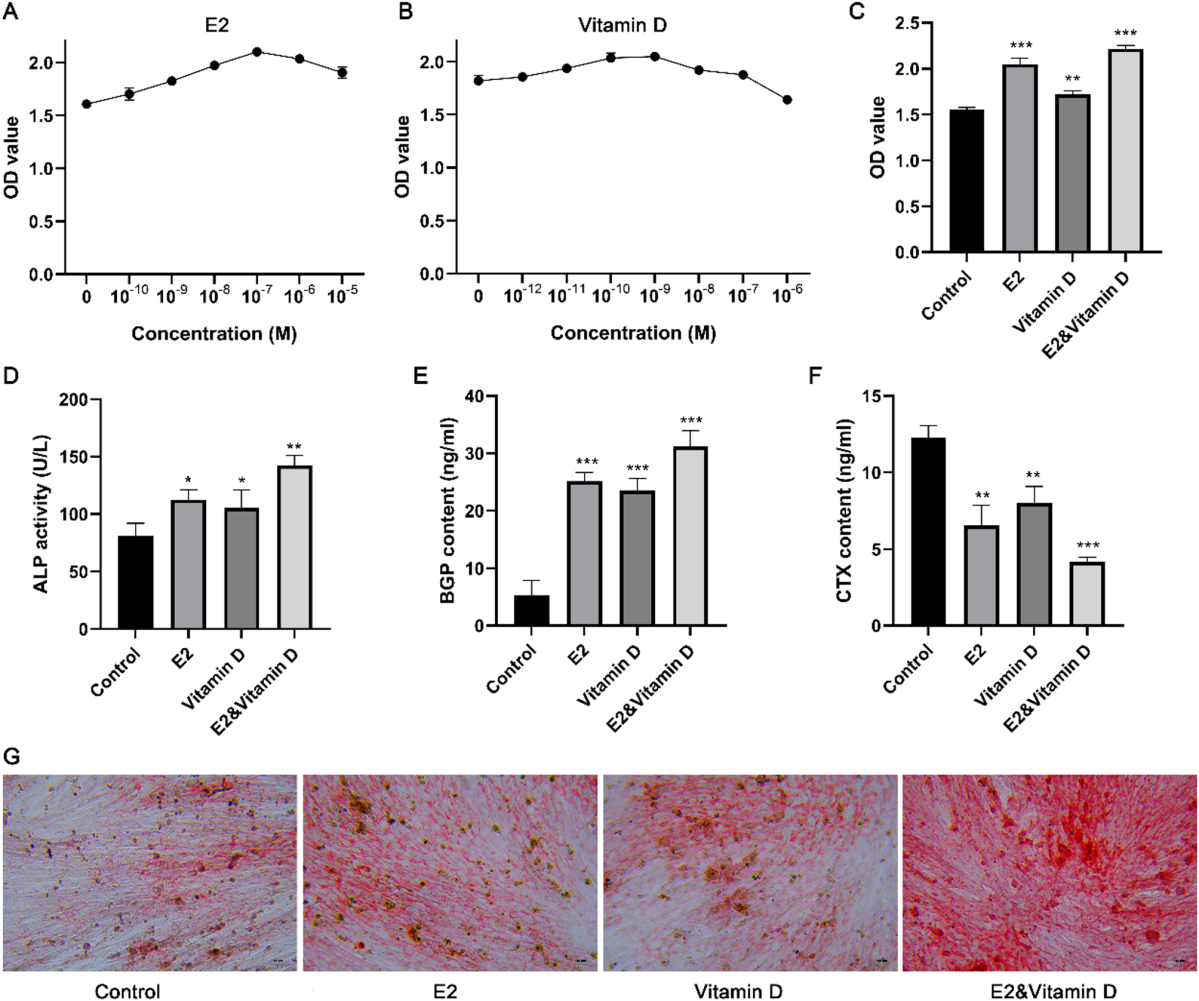
The effect of E2, vitamin D and their combination treatment on the proliferation and osteogenic differentiation of MC3T3-E1 cells. A-B: CCK8 assay showed the effect of different concentration of E2 and vitamin D on MC3T3-E1 cell proliferation. C: CCK8 assay showed the effect of optimal concentration of E2 and vitamin D and their combination treatment on MC3T3-E1 cell proliferation. D: The ALP activity after E2, vitamin D, and their combination treatments. E: The BGP content after E2, vitamin D, and their combination treatments. F: The CTX content after E2, vitamin D, and their combination treatments. G: The alizarin red staining results after E2, vitamin D, and their combination treatments. * *P* < 0.05, ** *P* < 0.01, and *** *P* < 0.001 compared to control group. Each value represents the average of triplicate measurements (*n* = 3).

To clarify the anti-osteoporotic effects of E2 and vitamin D, we examined the effects of different concentrations of E2 and vitamin D on MC3T3-E1 cell proliferation. Both E2 and vitamin D promoted cell proliferation at low concentrations, but high concentrations of these drugs inhibited cell proliferation (Fig. 2A-B). The optimal concentrations of E2 and vitamin D were determined to be 10^− 7^ M and 10^− 9^ M, respectively. Moreover, the E2 and vitamin D combination treatment exhibited a greater effect on cell proliferation than the individual drugs (Fig. 2C). ALP activity was also detected after treatment with E2, vitamin D, and their combination. ALP activity significantly increased after E2, vitamin D, and their combination treatment, with the combination treatment leading to the most pronounced increase in ALP activity (Fig. 2D). We further examined changes in bone metabolism markers (BGP and CTX) under different treatments. The results indicated that the BGP level in cell supernatants increased after treatment with E2, vitamin D, and their combination, whereas the CTX level decreased (Fig. 2E-F). Changes in BGP and CTX levels were most obvious after the combination treatment (Fig. 2E-F). Alizarin red staining results indicated that the red-calcified nodules containing mineral salts accumulated substantially after treatment with E2, vitamin D, or their combination (Fig. 2G). These data suggest that E2 and vitamin D synergistically promote MC3T3-E1 cell proliferation and osteogenic differentiation.

### miR-351-5p was downregulated after E2 and vitamin D combination treatment, the overexpression of which partially reversed the effect of the combination treatment on osteogenesis

**Fig. 3:**
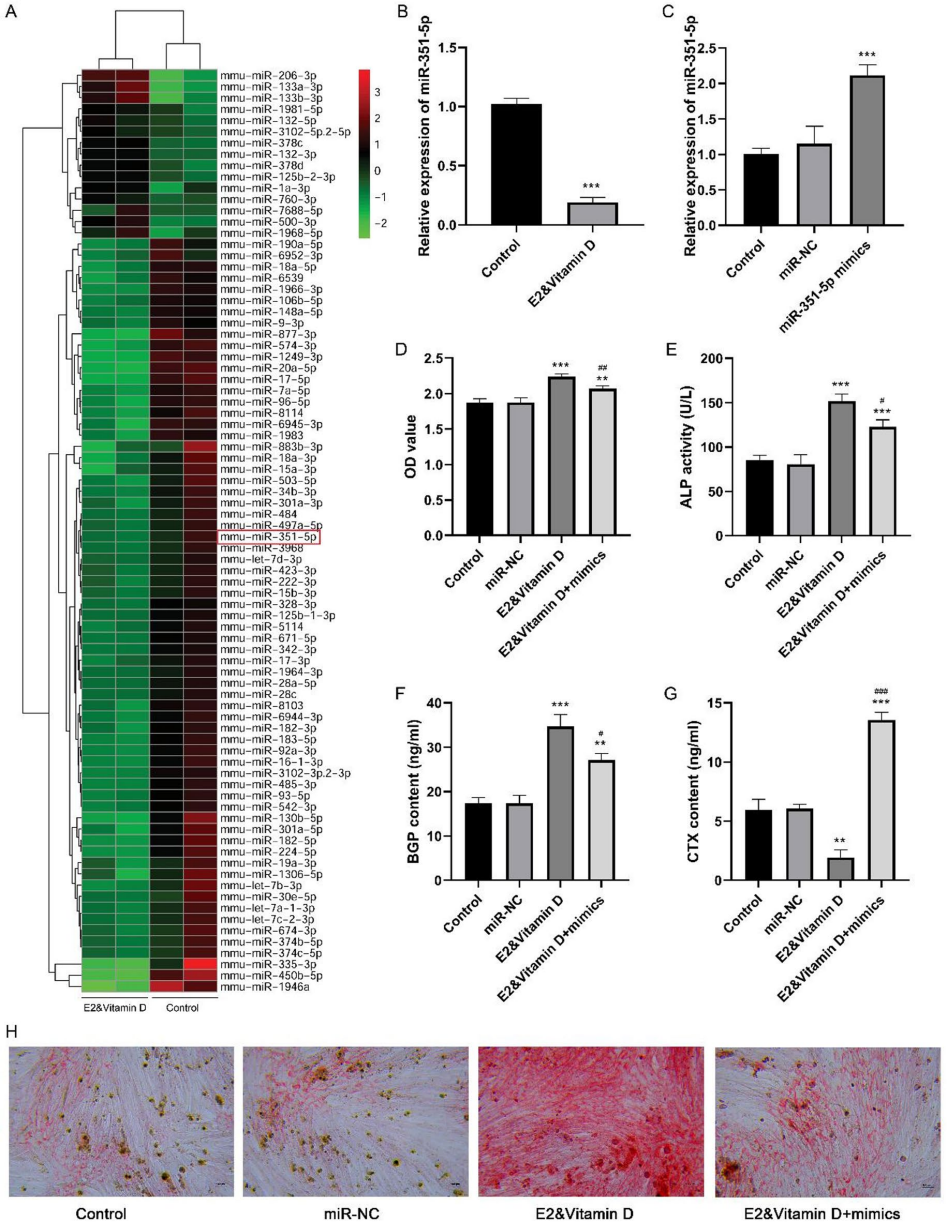
miR-351-5p was down-regulated after combination treatment of E2 and vitamin D, whose overexpression partially reversed the effect of combination treatment on osteogenesis. A: The expression heatmap of differentially expressed miRNAs between E2&vitamin D and control groups based on miRNA sequencing data. B: The miR-351-5p expression in E2&vitamin D and control groups. C: The miR-351-5p expression after transfection with miR-351-5p mimics and miR-NC. D: The MC3T3-E1 cell proliferation after E2&vitamin D and miR-351-5p mimics treatments. E: The ALP activity after E2&vitamin D and miR-351-5p mimics treatments. F: The BGP content after E2&vitamin D and miR-351-5p mimics treatments. G: The CTX content after E2&vitamin D and miR-351-5p mimics treatments. H: The alizarin red staining results after E2&vitamin D and miR-351-5p mimics treatments. * *P* < 0.05, ** *P* < 0.01, and *** *P* < 0.001 compared to control group. # *P* < 0.05, ## *P* < 0.01, and ### *P* < 0.001 compared to E2&vitamin D group. Each value represents the average of triplicate measurements (*n* = 3).

miRNA sequencing was performed to investigate the potential miRNAs ‌mediating the effects of E2 and vitamin D on osteogenesis. A total of 15 upregulated and 67 downregulated miRNAs were identified between the E2 + vitamin D and control groups. An expression heat map of the differentially expressed miRNAs is shown in Fig. 3A. Among these differentially expressed miRNAs, miR-351-5p was downregulated in the E2 and vitamin D groups compared with that in the control group. We further validated miR-351-5p expression in MC3T3-E1 cells after the E2 and vitamin D combination treatment. Consistent with the results of miRNA sequencing data analysis, miR-351-5p was dramatically downregulated in the E2 and vitamin D groups relative to that in the control group (*P* < 0.001, Fig. 3B). To further clarify the role of miR-351-5p, miR-351-5p was overexpressed in MC3T3-E1 cells by transfection with miR-351-5p mimics (*P* < 0.001; Fig. 3C). We found that miR-351-5p was overexpressed in MC3T3-E1 cells. Subsequently, we found that compared to the E2 and vitamin D groups, cell viability (Fig. 3D), ALP activity (Fig. 3E), and BGP content (Fig. 3F) were significantly decreased in the E2&vitamin D + mimics group, while CTX content (Fig. 3G) was significantly increased in the E2&vitamin D + mimics group. These findings indicate that miR-351-5p overexpression partially reversed the effect of the combination treatment on MC3T3-E1 cell proliferation and osteogenic differentiation. Moreover, the results of alizarin red staining demonstrated that miR-351-5p overexpression alleviated the accumulation of red calcified nodules containing mineral salts caused by the combination treatment (Fig. 3H).

### IRS1 was a target of miR-351-5p

**Fig. 4:**
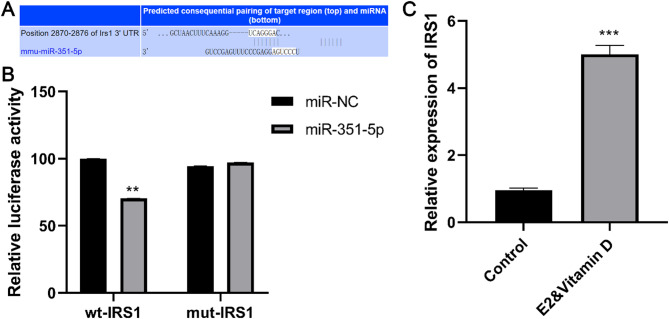
IRS1 was a target of miR-351-5p. A: The predictive complementary sequence of miR-351-5p and IRS1 predicted by Targetscan. B: Dual-luciferase report assay revealed the target relationship between miR-351-5p and IRS1. C: The IRS1 expression in the E2&vitamin and control groups. ** *P* < 0.01 and *** *P* < 0.001 compared to control group. Each value represents the average of triplicate measurements (*n* = 3).

To elucidate the regulatory mechanism of miR-351-5p, potential targets were predicted, and IRS1 was specifically identified. Figure 4A shows the predicted complementary sequence between miR-351-5p and IRS1, obtained using TargetScan. Subsequent dual-luciferase reporter assays revealed that the luciferase activity of wt-IRS1 was observably inhibited by miR-351-5p mimics (*P* < 0.01, Fig. 4B), indicating a target relationship between miR-351-5p and IRS1. Furthermore, IRS1 expression was markedly increased in the E2 and vitamin groups compared to that in the control group (*P* < 0.001, Fig. 4C).

### IRS1 knockdown partially alleviated the effects of the E2 and vitamin D combination treatment on osteogenesis

**Fig. 5:**
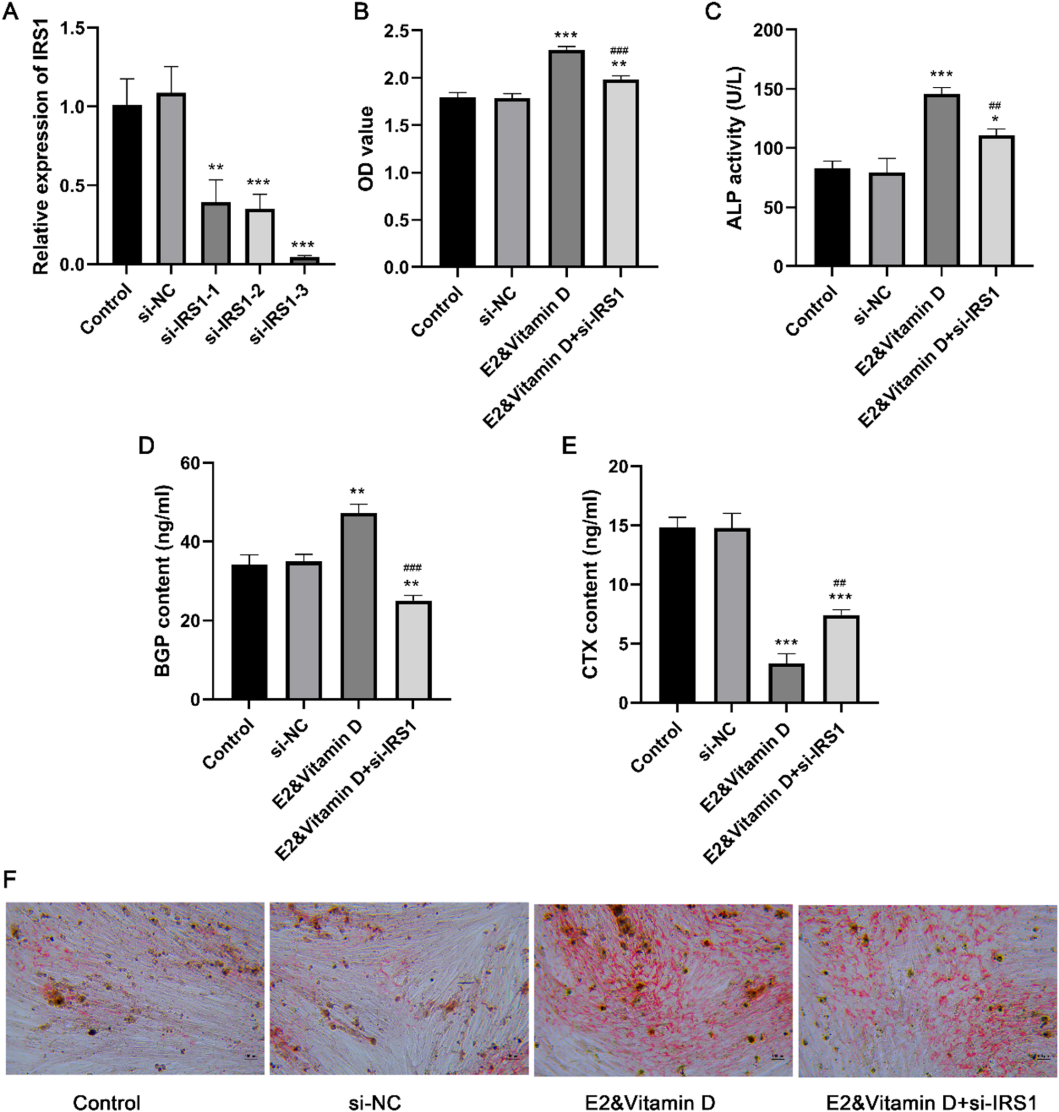
IRS1 knockdown partially alleviated the effects of combination treatment of E2 and vitamin D on osteogenesis. A: The IRS1 expression after transfection with si-IRS1-1, si-IRS1-2, and si-IRS1-3. B: The MC3T3-E1 cell proliferation after E2&vitamin D and si-IRS1 treatments. C: The ALP activity after E2&vitamin D and si-IRS1 treatments. D: The BGP content after E2&vitamin D and si-IRS1 treatments. E: The CTX content after E2&vitamin D and si-IRS1 treatments. F: The alizarin red staining results after E2&vitamin D and si-IRS1 treatments. ** *P* < 0.01 and *** *P* < 0.001 compared to control group. ## *P* < 0.01 and ### *P* < 0.001 compared to E2&vitamin D group. Each value represents the average of triplicate measurements (*n* = 3).

To explore whether IRS1 was involved in the combination treatment-induced proliferation and osteogenic differentiation of MC3T3-E1 cells, we knocked down IRS1 expression through transfection. IRS1 expression was markedly reduced after transfection with si-IRS1-1, si-IRS1-2, or si-IRS1-3 (*P* < 0.001; Fig. 5A). Among the three si-IRS1 sequences, si-IRS1-3 showed the highest transfection efficacy and was selected for subsequent experiments. We studied the effects of IRS1 knockdown on MC3T3-E1 cell proliferation and osteogenic differentiation following the combination treatment. Our findings revealed that compared with the E2&vitamin D treatment, the E2&vitamin D + si-IRS1 treatment resulted in significant reductions in cell viability (Fig. 5B), ALP activity (Fig. 5C), BGP content (Fig. 5D), and accumulation of red calcified nodules containing mineral salts (Fig. 5F), as well as an obvious increase in CTX content (Fig. 5E). These results suggest that IRS1 knockdown partially mitigated the effects of the combination treatment on MC3T3-E1 cell proliferation and osteogenic differentiation.

### Overexpression of IRS1 partially attenuated the effect of miR-351-5p overexpression on osteogenesis and the mTOR/NFκB signaling pathway under E2 and vitamin D combination treatment

**Fig. 6:**
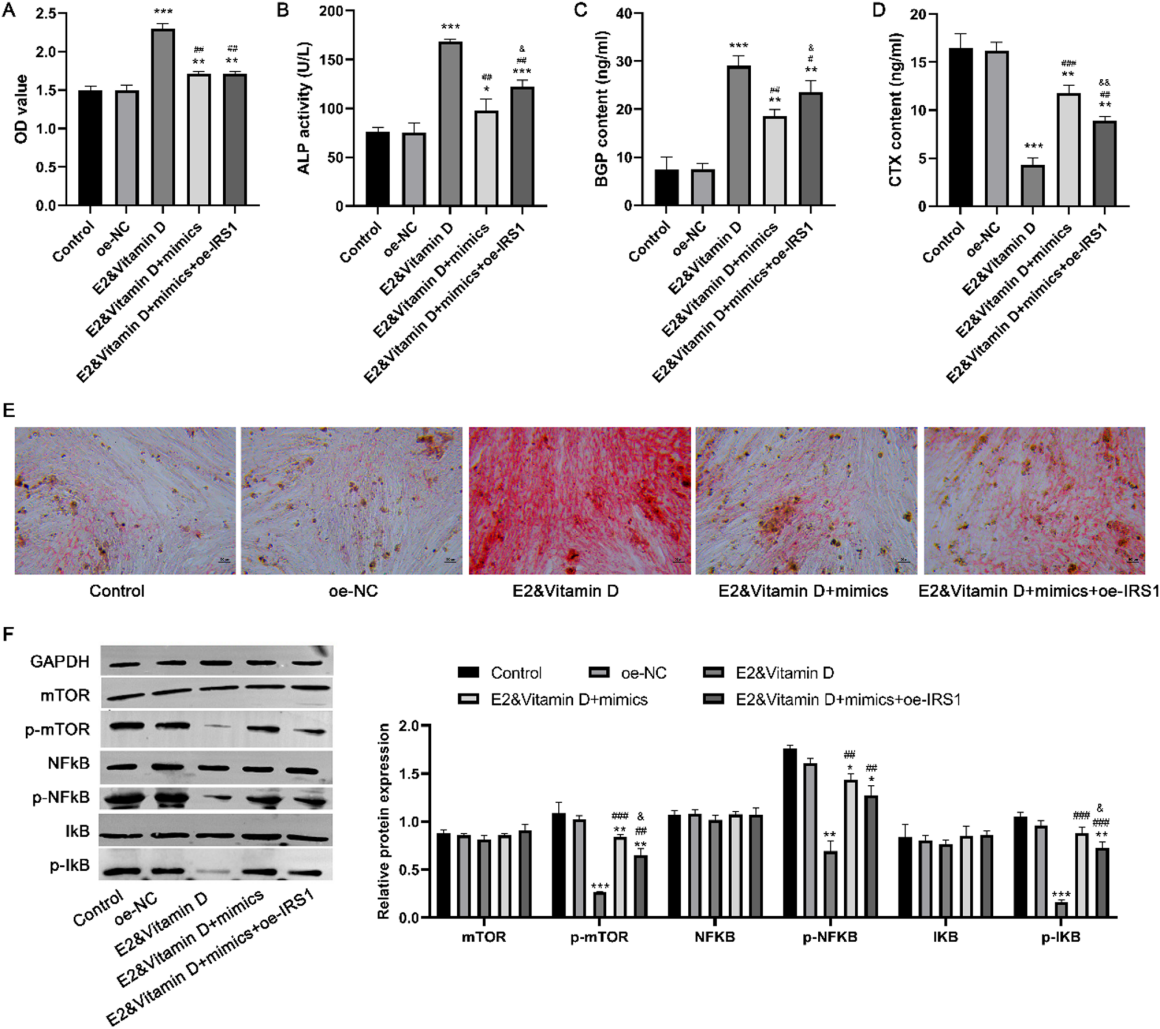
Overexpression of IRS1 partially attenuated the effect of miR-351-5p overexpression on osteogenesis and the mTOR/NFκB signaling pathway under E2 and vitamin D combination treatment. MC3T3-E1 cells were treated with E2&vitamin D and transfected with miR-351-5p mimics and oe-IRS1. A: The MC3T3-E1 cell proliferation of different groups. B: The ALP activity of different groups. C: The BGP content of different groups. D: The CTX content of different groups. E: The alizarin red staining results of different groups. F: The expression of the mTOR/NFκB signaling pathway-related proteins in different groups. * *P* < 0.05, ** *P* < 0.01 and *** *P* < 0.001 compared to control group. ^#^ *P* < 0.05, ^##^ *P* < 0.01 and ^###^ *P* < 0.001 compared to E2&vitamin D group. ^&^ *P* < 0.05 and ^&&^ *P* < 0.01 compared to E2&vitamin D + mimics group. Each value represents the average of triplicate measurements (*n* = 3).

To confirm whether IRS1 is a functional target of miR-351-5p, we investigated whether IRS1 overexpression could reverse the effect of miR-351-5p overexpression on osteogenesis under the combination treatment. Cell viability did not significantly differ between the E2&vitamin D + mimics and E2&vitamin D + mimics + oe-IRS1 groups (Fig. 6A). Significant increases in ALP activity (Fig. 6B) and BGP content (Fig. 6C), accumulation of red calcified nodules containing mineral salts (Fig. 6E), and a decrease in the CTX content (Fig. 6D) were observed in the E2&vitamin D + mimics + oe-IRS1 group compared with the those in the E2&vitamin D + mimics group, confirming that miR-351-5p played key roles in osteogenesis under the combination treatment via targeting IRS1. Moreover, mTOR/NFκB signaling pathway-related proteins were detected after different treatments (Fig. 6F). Consequently, the expression levels of p-mTOR, p-NFκB, and p-IκB significantly decreased in the E2&vitamin D group compared to those in the control group, indicating that the combination treatment inhibited the activation of the mTOR/NFκB signaling pathway. Additionally, the expression levels of p-mTOR, p-NFκB, and p-IκB dramatically increased in the E2&vitamin D + mimics group compared with those in the E2&vitamin D group, suggesting that miR-351-5p overexpression reversed the effect of the combination treatment on the mTOR/NFκB signaling pathway. Furthermore, IRS1 overexpression in the E2&vitamin D + mimics + oe-IRS1 group could partially attenuate the effect of miR-351-5p overexpression on the expression levels of p-mTOR, p-NFκB, and p-IκB under the combination treatment.

### E2 and vitamin D played a synergistic effect on preventing osteoporosis in vivo by inhibiting the mTOR/NFκB signaling pathway.

**Fig. 7:**
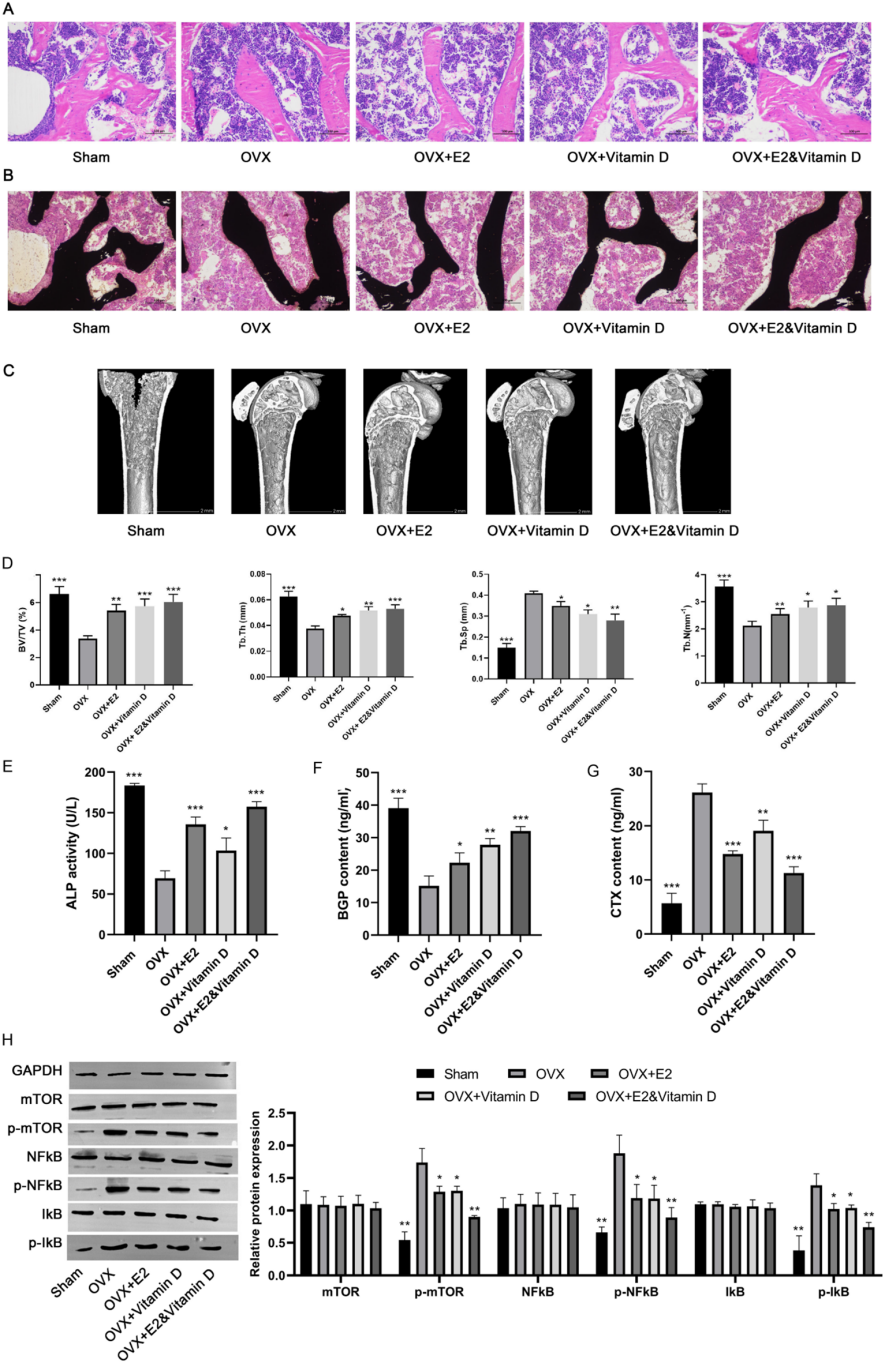
E2 and vitamin D could synergistically prevent osteoporosis in vivo via inhibiting the mTOR/NFκB signaling pathway. The mice were divided into sham (*n* = 8), OVX (*n* = 8), OVX + E2 (*n* = 8), OVX + Vitamin D (*n* = 8), and OVX + E2&Vitamin D (*n* = 8) groups. A: HE staining revealed the amount of trabecular bone of different groups. B: Von Kossa staining demonstrated the deposition of calcium nodules of different groups. C: Micro-CT analysis of bone loss of different groups. D: Quantitative analyses of bone structural parameters, including BV/TV, Tb.Th, Tb.Sp and Tb.N. E: The ALP activity of different groups. F: The BGP content of different groups. G: The CTX content of different groups. H: The expression of the mTOR/NFκB signaling pathway-related proteins in different groups. * *P* < 0.05, ** *P* < 0.01 and *** *P* < 0.001 compared to OVX group.

We further established an OVX mouse model of osteoporosis to investigate the effects of E2, vitamin D, and their combination in vivo. HE staining revealed that the amount of trabecular bone was significantly reduced in the OVX group compared to that in the sham group. After treatment with E2, vitamin D, and their combination, the decrease in the trabecular bone in OVX mice was alleviated (Fig. 7A). Von Kossa staining demonstrated a noticeable reduction in the deposition of calcium nodules in the OVX group compared to that in the control group. E2, vitamin D, and their combination increased calcium nodule deposition in OVX mice (Fig. 7B). Micro-CT analysis indicated that OVX mice exhibited significant bone loss compared with sham-operated mice, which was partially reversed after treatment with E2, vitamin D, and their combination (Fig. 7C). Subsequently, we conducted bone morphometric analysis of the femurs of mice, and the data showed that compared with sham-operated mice, the BV/TV, Tb.Th, and Tb. N of OVX mice were significantly reduced, while Tb. Sp was significantly increased. All parameters were partially reversed after treatment with E2, vitamin D, and their combination (Fig. 7D). Moreover, a remarkable decrease in ALP activity (Fig. 7E) and BGP content (Fig. 7F) and an evident increase in CTX content (Fig. 7G) were observed in OVX mice relative to sham-operated mice, which were partially attenuated after treatment with E2, vitamin D, and their combination. Notably, the combination treatment resulted in the most pronounced alleviation of these indicators in OVX mice. Furthermore, the expression levels of p-mTOR, p-NFκB, and p-IκB were dramatically increased in OVX mice, which were partially reversed after treatment with E2, vitamin D, and their combination, with the combination treatment showing the most pronounced effect (Fig. 7H). These data suggest that E2 and vitamin D synergistically inhibited the mTOR/NFκB signaling pathway in OVX mice.

## Discussion

Osteoporosis is the most prevalent bone disorder among adults and poses a significant health risk to women with respect to morbidity and mortality^20^. With the intensification of global aging, osteoporosis has emerged as an increasingly prominent focus in international research^21^. Osteoporosis often causes bone and tissue damage. Using digital models and additive manufacturing technology, 3D printing can create high-precision bone implants to resolve bone defects^22^. Mesenchymal stem cells can differentiate into chondrocytes and secrete cytokines to maintain the chondrocyte phenotype and promote their proliferation and extracellular matrix composition. It also plays an immunomodulatory role in various immune cells exposed to damaged tissues or inflammatory factors, making it a promising option for cartilage repair^23^. Notably, both vitamin D and E2 exert protective effects against osteoporosis; nonetheless, the synergistic role of these two factors in preventing osteoporosis remains largely unexplored. In the present study, we found that E2 and vitamin D synergistically promoted MC3T3-E1 cell proliferation and osteogenic differentiation. miR-351-5p was downregulated after the E2 and vitamin D combination treatment, and miR-351-5p overexpression partially reversed the effects of the combination treatment on osteogenesis. IRS1 was a target of miR-351-5p. IRS1 overexpression partially attenuated the effect of miR-351-5p overexpression on osteogenesis and mTOR/NFκB signaling pathway under the combination treatment. In vivo experiments further revealed that E2 and vitamin D could synergistically prevent osteoporosis in OVX mice by inhibiting the mTOR/NFκB signaling pathway. These findings demonstrated the anti-osteoporotic effects and regulatory mechanisms of E2 and vitamin D.

Postmenopausal osteoporosis (PMOP) is associated with a decline in estrogen levels^24^. Estrogen plays a crucial role in preventing bone loss and maintaining proper bone remodeling by activating vitamin D receptors in osteoblasts and osteoclasts^25^. A positive correlation is observed between elevated E2 levels and increased BMD as well as a decreased risk of osteoporosis in patients with type 2 diabetes mellitus^26^. Furthermore, E2 and vitamin D levels are positively correlated in women with osteoporosis^27^. Vitamin D, a steroid hormone, directly affects osteoblasts and osteoclasts and interacts with non-skeletal tissues to maintain a delicate equilibrium between bone turnover and bone growth^28^. Consistent with previous findings that vitamin D and E2 exert a synergistic effect on metabolic syndrome^10^, we also found that vitamin D and E2 play a synergistic role in preventing osteoporosis, suggesting that the vitamin D and E2 combination treatment may represent a promising therapeutic strategy for osteoporosis.

Numerous studies have revealed that miRNAs play a role in the regulation of bone metabolism^29^. Li et al. confirmed that extracellular vesicles derived from mesenchymal stem cells promote chondrocyte proliferation and migration through the circHIPK3/miR-124-3p/MYH9 axis, inhibit chondrocyte apoptosis, and promote cartilage repair, thereby preventing the development of osteoarthritis^30^. Zhao et al. found that miR-381-3p is a potential target for therapeutic intervention in PMOP, as it inhibits osteogenic differentiation by targeting the KLF5/Wnt/β-catenin pathway, thereby exacerbating PMOP^31^. In this study, miRNA sequencing analysis was conducted to elucidate the mechanism of action of the E2 and vitamin D combination treatment, and miR-351-5p was identified as a differentially expressed miRNA. The in vitro experiments further verified that the miR-351-5p expression was reduced after the E2 and vitamin D combination treatment and that the increased miR-351-5p expression partially reversed the effect of the combination treatment on osteogenesis. miR-351-5p has been found to be involved in many diseases. For instance, miR-351-5p exacerbates intestinal ischaemia-reperfusion injury by augmenting inflammation and oxidative stress^32,33^; miR-351-5p enhances lipopolysaccharide-induced acute lung injury through suppressing AMPK^34^; miR-351-5p is involved in hippocampal neural progenitor cell death and may be a potential target for Alzheimer’s disease treatment^35^. Notably, miR-351-5p is also involved in dishevelled 2-regulated osteogenic differentiation in a hyperlipidemia environment^36^. Therefore, we conclude that miR-351-5p mediates the anti-osteoporotic effects of the E2 and vitamin D combination treatment.

As miRNAs regulate disease development by modulating target gene expression^12^, we explored miR-351-5p targets and identified IRS1. IRS1 is expressed in preosteoblasts and regulates osteoblast differentiation^37^. A previous study showed that high glucose regulates the migration, proliferation, and mineralization of osteoblasts through the miRNA-144-5p/IRS1/AKT axis^38^. IRS1 acts as a critical molecule in the intracellular signaling of IGF1 and insulin, both of which have potent anabolic effects on bone metabolism. In addition to promoting bone formation and mineralization, IRS1 may also contribute to bone resorption, thereby controlling bone turnover^39^. Guo et al. revealed that IRS1 modulates bone formation by regulating collagen Iα2 expression^40^. Miao et al. further confirmed IRS1’s role in bone repair during diabetic osteoporosis^38^. In our study, IRS1 overexpression partially attenuated the effects of miR-351-5p overexpression on osteogenesis following E2 and vitamin D combination treatment. Thus, we speculate that the E2 and vitamin D combination treatment may exert preventive effects on osteoporosis by modulating the miR-351-5p/IRS1 axis.

The pathophysiology of osteoporosis is multifaceted, and the mechanism targets of mTOR and NF-κB pathways have become key factors for the regulation of bone homeostasis in countless cellular processes^41,42^. We conducted mechanistic studies to further investigate whether mTOR/NFκB pathway contributes to the anti-osteoporotic effects of combination treatment. The mTOR/NFκB pathway has been reported to be involved in mediating osteogenesis^43^. By inhibiting the mTOR/NFκB signaling pathway, timosaponin BII effectively activates the autophagy of osteoblasts, leading to an improvement in osteoporosis caused by hyperglycemia^44^. These findings collectively highlight the critical involvement of the mTOR/NFκB pathway in the pathogenesis of osteoporosis. Our experimental data demonstrated that the combination treatment inhibited the mTOR/NFκB pathway both in vitro and in vivo. It can be speculated that the inhibition of mTOR/NFκB pathway may be a pivotal mechanism underlying the efficacy of the E2 and vitamin D combination treatment in preventing osteoporosis.

This study confirms that E2 and vitamin D synergistically ameliorate osteoporosis by inhibiting the miR-351-5p/IRS1 axis and the mTOR/NFκB pathway. Despite the fundamental differences in the pathological mechanisms of osteoporosis and bone tumors (systemic bone homeostasis imbalance versus local malignant proliferation), the interplay between bone metabolism-related pathways and the complementarity of therapeutic strategies merits further exploration. First, at the molecular level, PTHR1 in osteosarcoma facilitates tumor cell invasion by modulating the AGT/CCL9 axis^45^. In our study, we observed that inhibition of the mTOR/NFκB pathway enhances osteogenic differentiation, suggesting that the same signaling node may exert opposite functions in different bone diseases, necessitating precise intervention to mitigate off-target effects. Second, in terms of therapeutic strategies, the cost-effectiveness trade-off between denosumab and zoledronic acid in giant cell bone tumors provides important insights for this study^46^. E2 and vitamin D combination treatment, in conjunction with existing antiresorptive agents, may synergistically enhance efficacy through multi-target approaches. However, systematic assessments of long-term safety and economic implications are required. Furthermore, the application of nanotechnology in the targeted delivery to bone tumors offers a technological translation pathway for osteoporosis treatment^47^. By developing bone-targeted nanocarriers loaded with E2 or miR-351-5p inhibitors, it is possible to significantly enhance the accumulation of drugs in the bone tissue and reduce systemic toxicity, thereby advancing the precision of osteoporosis treatment.‌.

This study confirms that the E2 and vitamin D combination treatment may be a promising anti-osteoporotic treatment strategy. However, this study had some limitations. First, it is unclear whether E2 and vitamin D play roles in other signaling pathways. Future studies should systematically explore additional molecular targets of E2 and vitamin D in the pathogenesis of osteoporosis. Second, the experimental scope was limited to cellular and murine models, and clinical validation was not conducted. Consequently, large-scale preclinical trials, followed by gradual clinical-phase studies, should be conducted to validate these findings and establish translational foundations for the E2 and vitamin D combination treatment.

## Conclusions

Our findings demonstrated that E2 and vitamin D exhibited synergistic efficacy in preventing osteoporosis by modulating the miR-351-5p/IRS1 axis and inhibiting the mTOR/NFκB signaling pathway. E2 and vitamin D combination treatment may be a promising strategy for osteoporosis treatment.

## Electronic supplementary material

Below is the link to the electronic supplementary material.

Supplementary Material 1

## Data Availability

The datasets used and/or analysed during the current study available from the corresponding author on reasonable request.
