# Supplementary material for: Estradiol and vitamin D exert a synergistic effect on preventing osteoporosis via the miR-351-5p/IRS1 axis and mTOR/NFκB signaling pathway
Source: Sci Rep. 2025 May 28;15:18678. doi: 10.1038/s41598-025-02808-z (PMC12119810; doi:10.1038/s41598-025-02808-z)

Figure S1 - full length gel images (GAPDH-1)


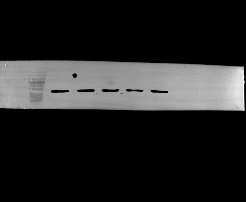


Figure S2 - full length gel images (GAPDH-2)


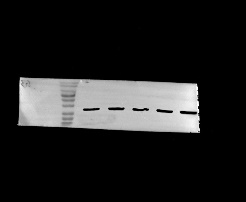


Figure S3 - full length gel images (GAPDH-3)


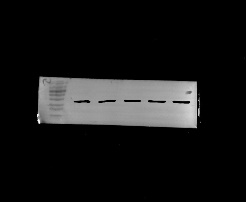


Figure S4 - full length gel images ( IKB-1)


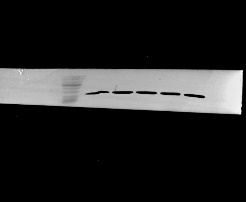


Figure S5 - full length gel images ( IKB-2)


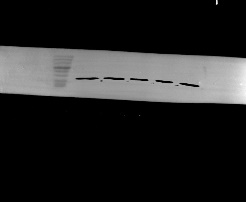


Figure S6 - full length gel images ( IKB-3)


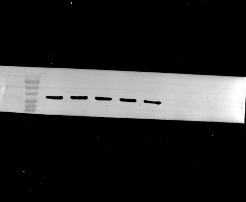


Figure S7 - full length gel images ( MTOR-1)


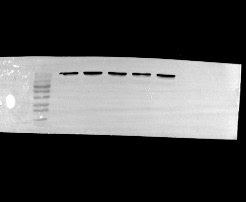


Figure S8 - full length gel images ( MTOR-2)


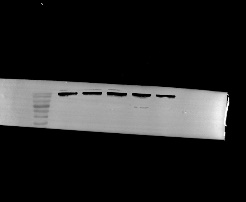


Figure S9 - full length gel images ( MTOR-3)


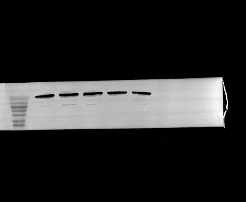


Figure S10 - full length gel images (NFKB-1)


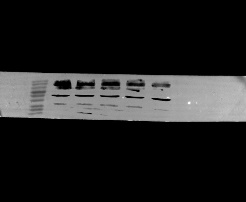


Figure S11 - full length gel images (NFKB-2)


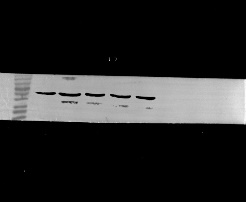


Figure S12 - full length gel images (NFKB-3)


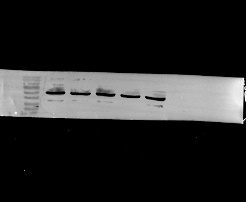


Figure S13 - full length gel images (P-IKB-1)


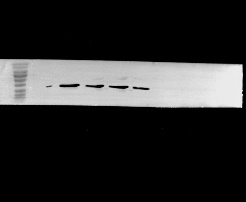


Figure S14 - full length gel images (P-IKB-2)


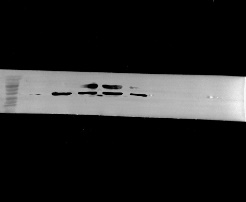


Figure S15 - full length gel images (P-IKB-3)


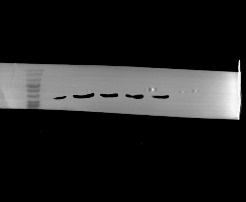


Figure S16 - full length gel images (P-MTOR-1)


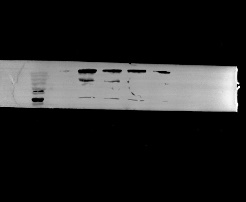


Figure S17 - full length gel images (P-MTOR-2)


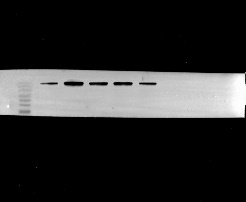


Figure S18 - full length gel images (P-MTOR-3)


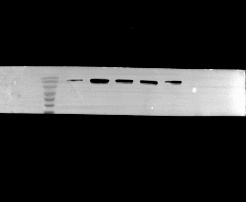


Figure S19 - full length gel images (P-NFKB-1)


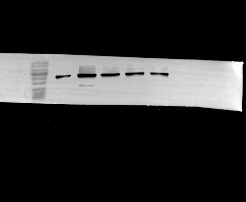


Figure S20 - full length gel images (P-NFKB-2)


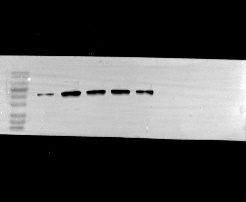


Figure S21 - full length gel images (P-NFKB-3)


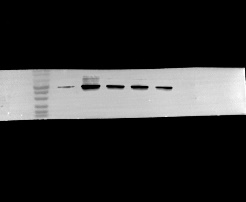

Supplement: Supplementary file 1 — Supplementary Material 1 [file 41598_2025_2808_MOESM1_ESM.docx]
